# A Hand-Book of Diseases of Women

**Published:** 1890-07

**Authors:** 


**A Hand-Book of Diseases of Women**; including Diseases of the Bladder and Urethra. By Dr. F. WINCKEL, Professor of Gynecology, and Director of the Royal University Clinic for Women in Munich. Authorized translation. Edited by Theophilus Parvin, M.D. Second edition. Revised and enlarged with 150 illustrations. Philadelphia: P. Blakiston, Son & Co., 1012 Walnut street. 1889.

This work of Professor Winckel became well known to the profession through the first edition thereof, hence we need not speak *in extenso* of this second one from the first German edition. A new section has been added upon Diseases of the Female Urethra and Bladder, in order to bring it forward to the line occupied by other similar treatises. This section has been added by the editor, Dr. Parvin, though it is chiefly a translation of Professor Winckel's monograph on this subject, the second edition of which was published in 1885. The editor has used his discretion as to which parts of the monograph to omit, and has made some of his own additions thereto, that are enclosed in brackets. The translator, Dr. J. H. Williamson, of Allegheny, Pa., has done no small part in making the work interesting through a clear and smooth translation. We are glad to possess such a valuable addition to our library, and congratulate author, editor, translator, and publisher on the valuable service they have each and all done the profession in bringing out such excellent literature.

